# ‘Baby mamas’ in Urban Ghana: an exploratory qualitative study on the factors influencing serial fathering among men in Accra, Ghana

**DOI:** 10.1186/s12978-023-01585-0

**Published:** 2023-03-01

**Authors:** Rosemond Akpene Hiadzi, Jemima Akweley Agyeman, Godwin Banafo Akrong

**Affiliations:** 1 Department of Sociology, University of Ghana, Accra, Ghana; 2 School of Management and Economics, University of Electronic Science and Technology of China, Chengdu, 611731 China

**Keywords:** Serial fathers, Family, Multiple partner fertility, Qualitative approach, Ghana

## Abstract

**Background:**

Biological fathering, especially in patrilineal societies, was traditionally acceptable only in the context of marriage to the mother of the child. Many men were polygynous, often staying in one household with all their wives and children. However, this phenomenon has been on the decline in recent times, mainly due to Christianity, which encourages monogamy while frowning on polygyny. The Ghanaian family has for the past few years been undergoing changes due to migration, urbanization, and industrialization. With an increase in non-marital births and the dissolution of marital unions, multi-partner fertility is likely to increase. Contemporary Ghanaian perspectives on the circumstances that lead men to engage in paternal multi-partner fertility, otherwise referred to in this study as serial fathering, are scanty, hence this study examines the factors that lead to serial fathering among Ghanaian men.

**Methods:**

The study employed the qualitative method, using in-depth interviews with twenty (20) serial fathers and a focus group discussion with seven (7) women.

**Results:**

It was found that factors such as the attitude of women in relationships, the duolocal post-marital residential pattern, and the age at first birth are some of the reasons why some men father children with multiple partners.

**Conclusion:**

The study concludes that both situational and personal factors account for the phenomenon of serial fathering amongst men in Prampram, Ghana, and these factors bring about distinctions in serial fathering as occurring either within or outside of marriage.

## Plain Language Summary

In Ghana, men are increasingly having children with numerous women, whom they refer to as their "baby mamas." Contemporary Ghanaian viewpoints on the factors that motivate males to engage in this behavior are minimal. Consequently, this study examines the factors that predispose Ghanaian men to father children by multiple women. In-depth interviews with twenty (20) serial fathers and a focus group discussion with seven (7) women from Prampram, a suburb of Accra, Ghana, were utilized in this qualitative study. The results of the study show that men had children with different women due to the negative attitudes of women, which pushed them to frequently change partners. The lack of a friendly relationship with in-laws, duolocal residence patterns, the non-observance of marriage rites, the young age of the first birth, and a continual need for companionship and sexual satisfaction were all factors that contributed to the practice. If these factors are addressed, it is anticipated that the prevalence of "baby mamas" in Ghanaian society will decrease significantly. This study also shows that family therapists and counselors should consider involving male clients' spouses when counseling them about having many "baby mamas."

## Background

A man who fathers children with more than one woman consecutively is typically referred to as a "serial father." This frequently results in having several sexual partners, which is challenging for the majority of mothers who end up having children with such men since they end up being referred to as "baby mamas." The phrase "baby mama" is applied to mothers who are not married to the child's father. Africa is not exempt from this; it has evolved into a universal norm over time. Studying serial fathering, which is also linked to having multiple partners, requires obtaining relationship data for all births, which is not normally obtained when conducting fertility studies [1, 2]. As a result, the study of serial fathering is a relatively new field of study in Africa because there is limited data [3].

According to Yeboah et al. [4] and Nyarko and Potter [5], serial fathering is on the rise regardless of whether or not a man has lost his spouse. Throughout many parts of the world, divorce and having many children have been linked to having multiple fathers [6]. Serial fathering is distinct from polygamy, which is the practice of marrying many spouses simultaneously. Serial fathering can occur without simultaneously marrying multiple partners, but only after a divorce or separation. However, given the difficulties that come with serial fathering, it has emerged as a crucial field of research for academics, as it is crucial to look at the factors that lead men to have several partners and consequently become serial fathers. This is because prior research has suggested that it is difficult for men who have children with numerous women to provide for the financial and social demands of all the families [7]. Typically, children from past relationships suffer when more resources are allocated to the current relationship [8].

Most research on serial fathering has been undertaken in Europe and the US, whereas few have been conducted in Sub-Saharan Africa, although this is a developing phenomenon. According to Guzzo and Dorius [2], multiple partnerships are being studied in the US because they may affect parental, child, and family wellness. In addition, they emphasized that the first step in studying a novel social phenomenon is to characterize and assess its prevalence. In the case of multiple partnerships, this is challenging because most data sources were not built to explore them. Petren [9] also assessed the relationship between paternal multiple partners (having children with two or more partners) and environmental chaos indicators among unmarried, non-resident fathers. Multiple paternal partners are related to environmental instability but not social support. Indicators include relationship insecurity, residential instability, job insecurity, and financial difficulties.

The study of multiple-partner relationships is, in accordance with Candia and Kisangala [10], a relatively new area of study. Their study examined male multiple-partner fertility in Uganda. According to their survey, 42% of males had multiple children. Age, being Muslim, and being divorced or separated increased the risk of multiple partner fertility, although being in the West, having a first sexual encounter after 19, and being married or cohabiting decreased it. There was a correlation between the number of wives or partners and lifetime sex partners and the risk of having numerous partners. Anecdotal evidence from Ghana suggests that men have children with multiple partners, (who they may or may not be married to) in turns and one frequently hears men (particularly in urban and peri-urban areas) refer to the existence of a "baby mother" or "baby mama". Socioeconomic factors, such as the expectation that men will provide their nuclear families with shelter, food, clothes, healthcare, education, etc., may contribute to this phenomenon [11]. One typical community where this practice can be found is, amongst the people of Prampram. Men were permitted to be polygynous in traditional Ghanaian society, as well as in other African communities, and they often lived in a single compound with all of their wives and children [12, 13]. Before marrying the second or subsequent wife, the man often asks for the first wife's consent and pays her a "pacification price"[14, 15]. Children also play an important role in such unions as they serve as social capital for their parents [16].

But recently, monogamy has replaced polygamy as the most socially and religiously acceptable option. However, men are still perceived as having children successively with various partners. Men opt to cohabitate or get married to one lady and have one or more children with her. These men move on to new wives when their marriages end. This occurrence is prevalent among urban and peri-urban residents. Whether or not they were married, men might still father children outside of their current families. However, there is a limited study on the factors that influence males to have multiple partners. Thus, the current study examines the factors that influence serial fathering in Accra, Ghana. This study is significant for two primary reasons. It is most likely the first study in modern Ghana to have addressed this significant gap in the sociology of the family literature in Ghana, and it is likely to be the only one. Second, concerning Dangme's marriage and childbearing practices, the findings also offer a cultural viewpoint.

The remaining sections are as follows: The literature is discussed in “Literature review” section, and the methodology employed for the study is presented in “Methods” section. “Results” section presents the study findings, and “Discussion” section discusses them. In “Conclusion” section, a conclusion is drawn.

## Literature review

### Masculinities in Ghana

A limited but expanding body of historical, ethnographic, and anthropological research in Ghana has attempted to comprehend gender in numerous domains of life before, during, and after colonization. Prior to colonial control, the dominant school of thought contends, the Ghanaian idea of gender was established as complementary between men and women, although boundary maintenance remained prominent and pervasive [17, 18].

Miescher [19] examined the changing meaning of becoming a man in modern Africa through the life stories of eight senior men. In addition, the report concentrated on the ideals and expectations that formed around prominent men in their respective communities when Ghana became an independent nation. How the men navigated complex social and economic transformations and dealt with their increasing obligations and responsibilities as leaders in their kinship groups, churches, and schools was also probed. Nevertheless, it must be acknowledged that Miescher not only explored masculinity and ideals of male behavior but also provided a new perspective on African men in a century of transformation. In addition to community standards, the findings indicate that missionaries and other colonial officials had a significant impact on notions of men and masculinity. The study's findings also indicate that the transition to manhood and a position of power, seniority, authority, and leadership was not always welcomed or straightforward.

Dery and Akurugu [20] acknowledged that there is limited academic research on how fathers themselves construct and represent masculinity in Ghana, although there is a growing debate among feminist scholars about how fathers often socialize their male children to aspire to embody particular values and behaviors. Men in rural northwestern Ghana are likely to embody hybrid masculinities where traditionally hegemonic masculine ideals—such as men being regarded as independent breadwinners—and contemporary gender-conscious norms—such as men as supportive presiding fathers—coexist. In subtle ways, however, the hybridization of masculinity both challenges and reinforces patriarchal gender arrangements. By maintaining a keen interest in their heteronormative breadwinning role as a model of masculinity, educated and gainfully employed men are critical of patriarchal norms that may be destructive to feminist discourses, but their depictions of masculinity indirectly strengthen male hegemony in marriage relationships. Also, their results show that there is a lot of ambiguity in how men define themselves as feminist allies by supporting gender equality, but none of them openly asks why women can't also be breadwinners.

According to Esson et al. [21], research in geography and allied fields on gendered experiences in Africa has struggled to comprehend the nuances that comprise the lifeworlds of young men. Their study examines the thoughts, actions, and experiences of male youth in Ghana through the lens of Guru's popular song "Boys are Tired," which is influenced by urban studies theories that demonstrate how music can be used to explore social dynamics in Africa. Based on interviews and focus groups with young people in Accra, the empirical findings and analysis demonstrate how the phrase "boys are tired" and the attitudes it evokes constitute a subversive critique and protest against the precariousness of contemporary Ghanaian urbanism. "Boys are tired" encourages the problematic (re)calibration of gender relations on patriarchal terms. In two novel ways, these insights advance discussions regarding the geographies of children and youth and gender relations. First, the vernacular of "tiredness" generates novel theoretical perspectives on a broader set of questions regarding youth agency and contemporary gender relations, specifically how young people are implicated in the reproduction of patriarchy. In doing so, their study identifies a troubling set of gender relations occurring in Accra that are conceptualized as "retaliatory patriarchy," which consists of three constituent elements: entitlement, resentment, and ignorance.

Dery [22] further acknowledges that critical studies on men and masculinities have gained considerable traction in feminist scholarship over the past several decades. However, the expanding focus of feminist scholarship has been on how individuals with male bodies construct, negotiate, and express masculine identities. Despite this growing interest, there has been insufficient research into how rural Ghanaian men construct and negotiate their masculinities in intimate relationships. The findings show that dominant notions of masculinity offer a broad context for understanding the narratives, negotiations, and experiences of intimate partner violence of the participants. The findings also suggest that father figures play significant roles in shaping and enforcing the conformity of their sons to traditionally hegemonic masculine ideals. These hegemonic ideals are so deeply ingrained in the larger social fiber that questioning them is rarely imaginable, thereby stifling alternative discourses. Even more convincingly, this study suggests that fathers shape their sons into "men" who emulate their own actions. It is instructive that fathers experience pride when their sons can live up to hegemonic masculine norms. The significance of these findings lies in their immediate revelation of the range of implicit and explicit cultural messages, proverbs, and metaphors that fathers use to define "desirable" and "undesirable" masculinity for their sons. In light of these findings, it is essential to emphasize that fathers' rigid enforcement of problematic gender identities can have negative effects on their sons, on women, and society as a whole. Importantly, when fathers strictly raise their sons and then approve of their unbending behavior, they make it harder for boys to find other ways to be men.

### Fatherhood and fatherhood roles

Modern fathers must supply, guide, help, and nurture. These positions can strain men's relationships with their partners, their sense of purpose at work, and their self-confidence as responsible adults. Due to the social nature of fatherhood, perceptions of parental engagement have changed over time. The men's ties to their own fathers, the nature of those interactions, and the father's emotional availability all affect how they feel about fatherhood [23]. Men emulate their fathers' parenting styles [24]. According to Meyer [25], fathers' traditional roles have shifted from being moral teachers and mentors to being breadwinners, role models, and loving parents. Industrialization, economic instability and dislocation, labor market changes, and gender equality calls reportedly influenced these outcomes. In the past, fathers supported the family. Fathers now have a wider range of responsibilities, including involvement and child care, as a result of women having more access to the workforce. Depending on their socioeconomic situation, fathers negotiate these roles with their spouses [26, 27].

Lewington et al. [28] confirmed that many men find fatherhood to be both rewarding and challenging. Their study investigates the effect of contradictory parental responsibilities on men's identities and how they negotiate them. Conventional masculine conceptions of fatherhood were both upheld and rejected by fathers. Men discussed traditional and non—traditional parenthood, masculine roles, and becoming and being a father. In fathers' parenthood experiences, male discourses influenced their relationships, occupations, and involvement with childcare. This study shows how uncertainty influences the roles of contemporary fathers and how they oscillate between traditional and contemporary parenthood.

Global South parenting emphasizes the customs and experiences of motherhood [29]. A study examined how males are seen as fathers and how they affect young children's development in Botswana. The majority of fathers in Botswana feel their primary role is to manage the family's finances while raising children is the mother's responsibility. Men don't use support services because they lack parenting abilities. To promote father engagement, a public research agenda is recommended.

### Multiple partner fertility (MPF)

Two types of multiple partner fertility (MPF) exist. The first one takes place outside of marriage. This is typical among young fathers who are unmarried to their children's mothers. The other type of MPF is found in marriage. This type of MPF occurs when married males father children with other women outside of their partnerships [30, 31]. MPF is when an individual has biological children with multiple partners [32]. Given the socioeconomic correlations of MPF and the repercussions of entering MPF for individuals and families, understanding the prevalence of such homes is crucial. Thomson et al. [33] admit that multi-partner fertility accounts for over 20% of total fertility in the US. Countries with high rates of first births outside of wedlock also have high multi-partner fertility rates. Multi-partner fertility is more uniformly spread across older ages than single-partner fertility, which peaks in the early to mid-20 s. Although the possibility of multi-partner fertility has increased during the decades analyzed, the rates of multi-partner fertility have remained rather stable.

### Factors relating to fatherhood with multiple mothers

Numerous studies indicate that several variables are associated with fathering in families with multiple women, a phenomenon commonly referred to as "multiple partner fertility" (MPF). These factors include:

#### First-birth-age

Multiple partner fertility is correlated with the father's age at first birth. According to U.S. and U.K. studies [1, 30], men who start having children young are more likely to have children with different women. Young fathers may live with their children's moms. Because cohabitation is less permanent than marriage, there's a higher possibility of remarriage and more children [34, 35]. Young individuals may not be able to work and support their families. The inability to financially support children might lead to breakups and new partnerships. Black men between 35 and 44 living in poverty report having children with several mothers [36]. Fathers having two or more children from many relationships seem to be at a disadvantage. Matlakala et al. [37] reported that in South Africa, young men can become fathers as young as 17, but Swart et al. [38] concluded that in KwaZulu-Natal, young men can become fathers as young as 14. Unmarried young men's relationships are precarious. Children from a previous partnership reduce the likelihood of parents marrying, causing men to have more children outside of marriage [39, 40]. Couples are less likely to marry or live together if the father (but not the mother) has previous children [41]. Having children with several partners can cause friction and estrangement in a couple's relationship following the birth of a new baby.

#### Race and educational attainment

Fathers' race or ethnicity is also associated with multiple-partner fertility. Multiple partner fertility is more prevalent among African fathers, who are often polygynous and take satisfaction in the number of children they may have [42, 43]. Men with childless wives want fertile women. According to Kane et al. [44], in-kind support accounts for a quarter of total support. Children receive in-kind support of $60 per month on average. Multilevel regression analyses show that younger children with longer visiting hours, high school-educated fathers, and no substance abuse difficulties receive more in-kind care. However, children whose fathers lack stable jobs or are of African heritage receive more support in kind. Later qualitative research indicated fathers' incentives for in-kind contributions are relational, not monetary.

Men with more education were less likely to father children with more than one woman and were better informed about their reproductive choices [45]. They understand health better, are more likely to be married, and raise healthy children. They reduce child behavioral issues [46, 47].

#### A woman's natal count

Some believe that the number of children a man has with a single woman indicates whether or not he will have additional children. According to Nisén et al. [48], fatherhood decreases a man's fertility, but this does not prevent men from desiring larger families because they can build social capital. Men are more likely to have children with other women if they are married and their wives exhibit signs of infertility.

#### Fathers who report unintended pregnancies

Fathers who tell their spouses about unwanted pregnancies are more likely to have multiple relationships [49]. By revealing unexpected pregnancies, they hoped to avoid penalties in the future. Grindlay et al. [50] studied unexpected pregnancies in Accra, Ghana. Nearly half of females said their most recent pregnancy was unplanned, and most felt at risk for future unexpected pregnancies. Women were more likely to have an unplanned pregnancy if they had previously given birth, made their sexual debut between ages 8–14 instead of 20–24, or had 3–4 sexual partners rather than 1–2.

#### Men's income

The financial status of a man indicates whether he would father children with multiple women. Numerous studies show that men with higher incomes are more likely to marry and establish happy families [47, 51, 52]. Poor men have more children [53]. They reproduce with multiple partners to reaffirm their masculinity. Their relationships will be unstable, and they will likely have children outside of marriage.

### Marriage formation in Ghana

Many Ghanaians value marriage because it serves as the foundation for determining who will perform reproductive, economic, and noneconomic roles [54]. Additionally, Ghana's pro-family and pro-marriage ideology has repercussions for social relations, and among the various ethnic and linguistic groups, single women and men are frequently viewed differently from those who are married [55]. This is gradually encouraging the majority of Ghanaians to develop the practice of getting married young [56]. Amoateng [57] makes the additional claim that marriage is almost universal in Ghana and that having children is expected of couples. The family serves as the cornerstone of social structure, the main provider of old age security, and the primary caregiver for the young.

The Children's Act of 1998 and Ghana's Constitution from 1992 both specify the legal age of marriage and union formation as 18 years old and forbid these activities before this age [58]. Under Ghanaian law, the legal age to marry is the same for all, which respects both traditional and Islamic unions. The laws of Ghana are superior to any religious doctrine, although the country's constitution guarantees everyone the right to freedom of worship. Therefore, it is forbidden in Ghana to marry anyone under the age of 18, whether for religious or customary reasons. When both families of the woman and man are aware of the union, a man and a woman are generally accepted as husband and wife in Ghana. Additionally, the formation of marriages in Ghana serves to give couples companionship, a way to support one another, and a legal means of engaging in sexual activity and procreation [59]. Members of each family are checked for diseases that cannot be cured or spread, criminal records, violent behavior, respectability, occupation, and religious background. However, a potential spouse must also meet some important requirements before being accepted.

## Methods

### Study location

The study was undertaken in Prampram. It serves as the Ningo-Prampram District's capital in the Greater Accra Area. It has a population of 204,673 individuals [60]. In addition, Prampram has two major suburbs: Upper Town and Lower Town, which are made up of the four (4) communities of Lower East, Lower West, Kley, and Olowey. These communities are home to the four major clans, which include the "Larkpl3," who live in the Lower East and West, as well as the Kley, Anewey, and Olowey. Dangme and Ga are widely spoken there.

One significant reason for the choice of Prampram as the study area is that it is a peri-urban community. In other words, it has characteristics of both urban and rural settlements. According to Tach et al. [61], multiple-partner fertility, or serial fatherhood, is prevalent in peri-urban areas and more so among low-income fathers. Prampam is also a patrilineal society where a child born into the family belongs to the paternal agnatic kin. In addition, the residential pattern is duolocal. The woman prepares meals at her home and sends them to her husband’s home in the evening, where she passes the night. The man could also come and spend the night in the woman’s home occasionally. The women basically raised their children in a matrifocal setting. When boys reach puberty, they are sent to join their fathers on the male compounds, while girls remain with their mothers.

### Research design and setting

The study employed a qualitative exploratory research methodology based on phenomenology and the interpretivist paradigm. This research design typically collects participant data for analysis through in-depth interviews [62, 63] and focus groups [64]. The purpose of phenomenology is to characterize the meaning of concepts or phenomena from the perspectives of multiple individuals who encounter them [65, 66]. This research phenomenon was chosen because it allowed the researchers to examine in depth the factors that influence serial fathering among fathers in Prampram- Accra. The sociodemographic features of the participants and the factors influencing serial fathering in Prampram were among the issues investigated in this study. The exploratory research approach was equally relevant to the study because the researchers had a general notion about serial fathering that they wished to investigate, but there was no prior knowledge in the Ghanaian setting to explore it.

### Sampling and procedure

For the study, a sample size of 20 male serial fathers and 7 female participants was purposefully selected from Prampram. It excluded men who fathered children with a single woman and those who fathered children with many women but shared a residence with them and their children. For the purposes of this study, the women with whom the men had children were referred to as "baby mamas." The inclusion of women was undertaken to acquire a gendered viewpoint on the subject under study. In-depth interviews were conducted with the community's serial fathers, and a focus group included the women.

Due to the nature of their occupation, meetings were arranged with the identified serial fathers who agreed to participate in the study, and interviews were conducted at their residences and places of work (see Table 1). All participants were provided with information regarding the study, the intended use of the collected data, and the anticipated interview duration.

**Table 1:** Socio-demographic characteristics of respondents

Serial Fathers	Age	Current marital status	Occupation	Number of Children	Number of “Baby mamas”
John	41	Cohabiting	Mason	8	4
Adjei	18	Separated	Fisherman	3	2
Gabby	63	Married	Farmer	5	4
Daniel	65	Married	Fisherman	15	5
Joshua	53	Cohabiting	Driver	3	3
Mike	30	Married	Farmer	5	4
Simon	35	Married	Unemployed	5	4
Raman	19	Single	Okada Rider	6	3
Kwame	64	Married	Carpenter	4	2
James	32	Cohabiting	Carpenter	2	2
Kofi	24	Single	Barber	4	3
Baba	32	Cohabiting	Carpenter	3	3
Kojo	30	Single	Steel Bender	5	3
Sammy	31	Married	Okada Rider	12	3
Wise	45	Married	Driver	4	3
David	19	Single	Okada Rider	2	2
Kwesi	45	Cohabiting	Civil Servant	4	4
Paul	58	Separated	Tailor	6	3
Ali	55	Married	Civil Servant	5	2
Ayi	53	Cohabiting	Civil Servant	5	3
Women	Age	Current marital status	Occupation	Number of Children	
Martha	40	Cohabiting	Fish Monger	4	-
Abena	35	Separated	Hairdresser	3	-
Rejoice	32	Married	Trader	2	-
Abigail	38	Separated	Seamstress	4	-
Mary	33	Married	Baker	3	-
Rose	30	Separated	Trader	4	-
Grace	28	Married	Hairdresser	2	-

An in-depth interview guide was developed to gather data regarding the factors influencing serial fathering. The in-depth interview collected data on the socio-demographic characteristics of the respondents, while Section Two examined the factors that influenced their decision to have children with different women. The participant interviews were conducted in Dangme and Ga (Ghanaian dialects), which was convenient for both the researchers and the participants. Before the interviews commenced, all participants were provided with information about the study, the intended use of the collected data, and an estimate of the interview duration. A 45-min-to-1-h-long interview was conducted. All participants were consulted beforehand on the audio recording of the interview. Participants were requested to complete an informed consent form upon agreement. Throughout the study, the confidentiality of personal information was preserved. Accordingly, no personal identity was associated with the interviewees. In addition, the names used to present the analysis are all pseudonyms and do not correspond to the true identities of the participants. Due to the fact that the interviews were done in the local languages (Dangme and Ga), they were back-translated and transcribed into English.

Whenever necessary, field notebooks and interview tapes were used to fill the gaps discovered throughout the translation process. In order to verify audio recordings and transcribe interviews, peer debriefing and member checks were also employed. Before fieldwork began, ethical approval for the study was acquired from the Ethics Committee for Humanities (ECH) at the University of Ghana, Legon, with approval number ECH 085/18–19.

Using purposive sampling, 26 respondents were approached, but 6 of them declined, citing *"I am terrified." Oh, as for you guys, you are interviewing me so that you can send me to the Family Tribunal"* and *"I hope you won't give my response to the media."* With this feedback, the researchers utilized a gatekeeper to sample the remaining responders. This made the respondents more at ease, as the gatekeeper informed them that the study was conducted only for scholarly reasons.

The focus group was also held with seven (7) women in Prampram. This was conducted to determine the gender dimensions of fatherhood. It allowed the women to openly express their opinions on the topic under investigation and gave a woman's perspective on the issue. Several of the concerns examined were similar to those raised during the interview sessions. The primary purpose of the focus group discussion was to confirm the prior responses and identify any discrepancies that may have developed during the interviews and informal conversation sessions.

### Data analysis

The obtained data were analyzed thematically using NVIVO software version 12 to run the codes, categorization of themes, and subthemes that arose from the interview transcripts of the participants. The thematic analysis was conducted using both inductive and theoretical coding techniques. Inductive and theoretical coding approaches were utilized in the analysis since they assisted the researchers in identifying, analyzing, and presenting data patterns.

For inductive coding, the researchers began by reading the interview transcripts and paying close attention to relevant data concerns, which led to the development of descriptive codes. This was immediately followed by the identification of cluster themes, whose meanings were evaluated in the context of the study objectives and refined in light of the reviewed literature. Similarly, the reliability of the findings was ensured by contacting eight of the participants to confirm the reported results as part of the participant validation method.

## Results

### Research participants

In the study, there were a total of 20 male serial fathers and 7 female participants. As was discussed in “Methods” section, the inclusion of women was undertaken to obtain a gendered perspective on the phenomenon that was being studied. Table 1 provides a further presentation of the findings derived from the data that was gathered.

### Factors leading to fathering with different women

The findings of the study show that six major factors contribute to the prevalence of serial fathering among men in Prampram, and these factors are discussed in greater detail below.

### The attitude of women in relationships

This theme describes the various ways in which the women that the men in this study had fathered children with showed attitudes that were not favorable to them and thus caused them to move on to father children with other women. These unfavorable attitudes have been grouped into four sub-themes, namely: disrespect and non-performance of wifely duties; suspected infidelity; involvement in social vices; and maltreatment of stepchildren.

- *Disrespect and non-performance of wifely duties:* Some men got involved with other women and had children with them because, according to them, their first baby mamas were not submissive and did not perform their "wifely" roles to their satisfaction. According to Joshua:

*She did not respect me. If I ask her to cook for me, she will refuse. She did not wash my clothes. She kept our room untidy always. When I complain, she insults me very well. I could not contain her behavior, so I went for another woman and left the room for her. (Joshua, 53-year-old father of three (3) with three different women)*

*That woman was dirty and lazy. She will watch TV all day. Sometimes, when she is cooking, she leaves the food on the fire and goes to sleep. When I try to talk to her nicely about doing housework, she will ask me if I don’t have hands to also do the work. I realized she could not take care of me, let alone the children, so after having one child with her, I left the relationship. (Kojo, a 30-year-old father of five children with three different women)*

b. *Suspected infidelity*: A few fathers who decided to enter into new relationships with other women did so because they suspected their legally married wife was licentious. They often heard rumors from their family members that these women had been seen with other men in the neighborhood. As one respondent intimated:

*My wife was flirting. Anytime I came back from work, my sister told me that my friend was in our room watching TV. When I confront her, she denies it and calls me names. One day, I saw my wife in a very compromising position with a man. I decided to leave her and go for another woman. I am now with a different woman who just delivered a baby boy. (Ayi, 53-year-old father of five children with three different women).*

iii. *Involvement in social vices*: Alcoholism is one of the vices that discourage some fathers from staying in a relationship with their baby mamas. Alcoholism was seen as acceptable for men but not for women, especially married women. As such, although Simon loved to enjoy his bottle of alcohol, he could not accept similar behavior from his spouse. According to him:

*I went on to marry a fourth woman because my third spouse started drinking. I was surprised that a married woman could drink so much. I know that I also drink, but I could not understand how a woman could drink the way she did. The worst part was that, sometimes, she gave some of the alcohol to our 5-year-old son to also drink. I mean, how could she do that? (exclaims...) Teaching a child how to drink? No, I will not accept that. (Simon, 35-year-old father of five children with four different women)*

iv. *Maltreatment of step-children:* The results from the study also show that for some other men, their motivation to have children with multiple women stemmed from the maltreatment their current partners meted out to children they had with previous partners. Although at the onset of the new relationship, the women displayed love towards their stepchildren, they soon changed their attitude once they had children with the men and began to show preferential treatment towards their own children while maltreating their stepchildren. According to James,
  *Things began to change when I brought Sandra (my 9-year-old daughter from a previous marriage) to stay with us. I observed how my second wife, Mamle, tried to prevent Sandra from interacting with her daughter, Benedicta. Sandra was not allowed to touch any item bought by Mamle. I was not happy with what was happening in the house. I spoke about it to my wife, but it always ended up in a quarrel. The situation became worse by the day. It has gotten to the point where she will not even allow Sandra into the house when I am not at home. I have decided to end my relationship with Mamle because I love my daughter Sandra very much and I cannot afford to see her being maltreated by Mamle. (James, 32-year-old father of two children with two different women)*

### Strained in-law relationships

This theme describes how, according to the findings, some fathers were compelled to leave their first baby mamas for new relationships due to the constant quarrels and misunderstandings that ensued between their spouses and their family members. To them, such unhealthy relationships did not need to be perpetuated, and the best way to avoid them was to end such relationships since there was no end in sight to the strained relationships between their spouses and their family members. For some, it was their spouses who did not like their family members, as indicated in the quote below:

*I never thought I would have children with more than one woman. I grew up in a polygynous home. I saw how my late father had a hard time managing his home. Hardly a day passes by without one fight or another. Anytime my stepmother fought my mother, I was always sad. I vowed to marry only one wife. But things didn’t go as I planned. The first woman I courted was a thorn in my flesh. She always had issues with my mother. I tried my best to get her to stop, but it was not working. I eventually had to break up with her because I was tired of the fact that she always quarreled with my mother. I did not like it at all. (Gabby, 63-year-old father of five children with four different women)*

As the following quote shows, for some individuals, it was the members of their own families who did not get along with their spouses.

*My mother always had issues with the woman who had children with me first. She just did not like her. One day, when she came to visit me at my house, they nearly fought. I had no option but to let her go. The woman I am married to now was suggested to me by my mother. (Sammy, 31-year-old father of 12 children with three different women)*

For others, it was the spouse's family that did not accept or acknowledge them as spouses for their daughters. Kwame was forced to divorce his wife since his in-laws disapproved of their union from the beginning. According to his assertions,

*I had two children in my first marriage, but that relationship eventually ended simply because my in-laws never approved of my relationship with their daughter. They kept interfering in the marriage until we went our separate ways. The children stayed with their mother after the divorce. My ex-wife remarried, but I am able to visit her and the children anytime I feel like it. I am also married to another woman now. (Kwame, 64-year-old father of four children with two women)*

Whatever form these strained in-law relationships took and wherever they originated, it was viewed as a reason to end the partnership and seek out other partners with whom they might develop healthier in-law relationships.

### The Duolocal pattern of residence

This theme describes a cultural element existing among the Ga that was seen by both the men and women in the community as facilitating the incidence of serial fatherhood. In the interviews, twenty-five (25%) of the fathers who did not share the same residence with their spouses reported that they found it easy to get into other relationships based on this residential arrangement. It is a common practice in Prampram for couples to live apart, as is characteristic of the Ga-Adangme. The following narratives illustrate the theme:

*In my heydays, I slept with different ladies anytime I wanted because I was staying in my father’s house while my wife was also staying in her father’s house. I had money and I could take the women out. I only had to pick a fight with my wife whenever I wanted to be with a different woman. She will get so offended that she will refuse to bring my food and sleep in my house at night for even more than a week sometimes. Then bingo! I got the opportunity to explore and have fun. Those were the good old days. That made me have children with many women. (Daniel, 65-year-old father of 15 children with five different women)*

*We have a young men’s room in the family house. Once I get a girl who agrees to my proposal, I just send her there. My girlfriend is not aware of it. (Adjei, 18-year-old father of three children with two different women)*

*On days that my woman does not come to sleep in my house for one reason or another, I just go to town and get myself another woman. It is not too difficult to get a woman to sleep with you. Unfortunately for us, some come with pregnancies. (Wise, 45-year-old father of four children with three different women)*

This notion about the residential arrangement fueling the practice of serial fatherhood was corroborated by the women in the community during the focus group discussions as well. According to one discussant,

*My father married my mother, and they had three of us. I did not know my father till I was about 10 years old when he visited us at my mother’s father’s house. All along, my father had been living in his father’s family house. My mother told me that she used to spend the night at my father’s family house. Being a fishmonger, on nights of bumper catch, she had to stay all night to smoke the fish and so could not spend the night with my father at his family house. Unfortunately for my mother, she had the shock of her life when she found out that my father had impregnated another woman. That was the end of their marriage. Thinking of the incident now, I believe this residential arrangement made it easy for my father to cheat on my mother.* (*Abigail, 38-year-old,Separated)*

### Non-performance of marriage rites

In this section, the study shows how men who have children with women they are not married to or have not completed marriage rites within patrilineal descent systems have weaker social identities. Such men are not recognized by their would-be in-laws as legitimate husbands. Daughters are therefore requested by their family members, especially their fathers, to end their relationship with such men until they perform the marriage rites. The responses from some fathers indicated that their unions were not approved by would-be in-laws because of their non-performance of the required marriage rites. Thus, anytime they had a problem or disagreement in their relationships, the women were advised by their relatives to quit. According to the study's findings, one respondent responded by retracing how his relationship with his first baby mama ended, stating:

*We had been seeing each other for about three years, although I must admit I had not yet performed the necessary marriage rites for her. In the second year, she became pregnant. Initially, I was preparing to perform the marriage rites, but I could not get money for it. She became pregnant again. Hmm... Her family was asking me to perform the rites or risk losing her. Unfortunately, I still could not perform the rites, so eventually, her father asked her not to see me again. It was difficult for me, but being a man, I have also moved on. (Joshua, 53-year-old father of 3 children with 3 different women)*

A discussant from the female focus group believed that the men in the community were just not committed to having a relationship with a woman that would lead to marriage simply because they preferred to have multiple sexual partners. According to her,

*Men here don’t like marriage. They propose to you, and as soon as you agree, they start sleeping with you. When you become pregnant, they present two bottles of schnapps to your parents, and that is all. Before you know it, they are sleeping with other women and having children all over the place. I call them "yεnk⊃ nkoaa " (translated as "let us keep going"). (Martha, 40-year-old, cohabiting)*

This shows that it is somewhat simpler for males to seduce women without following the customary procedure of formally seeking the woman's hand in marriage from her family and then performing marital rites. This phenomenon pushes men (who may already be fathers) to have children with various women at various times.

### First-birth-age and related factors

The findings from the study also show that the early onset of childbearing facilitates multi-partner fertility among men in Prampram. About 10% of the fathers in this study stated that they started giving birth at an early age and at a time when they were not prepared to handle the responsibilities that came along with having children. The following narratives exemplify the theme:

*My first child was born when I was 17 years old. My parents were farmers, and I would go to the farm with them. Though I had wanted to be enrolled in school, it did not happen for me. I had a number of female friends. One day, I slept with one of them, and she became pregnant. My parents took responsibility for the pregnancy till she delivered. They warned me not to sleep with her again. By age 26, I realized I had three children. Hmmm... now I have 5 with 4 women. (Mike, 30-year-old father of five children with four different women)*

*While I was in secondary school, I impregnated one of my classmates and had a son (Isaac) with her. My mother took him and cared for him while I sent her money occasionally for his upkeep. After my polytechnic education, I got married by ordinance to Maku. It was a big wedding which was the talk of the town. We stayed in one of my father’s houses. After 8 years of marriage, I went to have a child with another woman in my village. My wife found out and filed for a divorce. (Ayi, 53-year-old father of five children with three different women)*

*I had my first child when I was seventeen years old. I was then in JSS Two. I had to stop my studies because my mother, who was then taking care of me, could not afford to take care of me, my girlfriend, and our baby. When she delivered, my mother asked her to come and stay in our house so that things would be easy for her. But that was where all the problems started... I saw that she wasn’t a wife material and decided to call it quits (Joshua, 53-year-old father of three children with three different women).*

Having a first child at a relatively young age (in this case, during adolescence) creates a situation in which men are unable to financially support their first baby mama and child, resulting in the breakup of the relationship. Because these men were young, they were not mentally ready to make a long-term commitment to their first baby mamas. In addition, the findings from the study show that several men fathered children with multiple women at an early age due to peer pressure. As stated by a respondent:

*I was exposed to sexual activities early. We were a group of friends who dared each other about the number of ladies we could sleep with. At the time, we had no idea it would result in pregnancies. The reality dawned on us when the ladies started getting pregnant for us. It was not easy for us. (Raman, 19-year-old father of six children with three different women)*

### The constant need for companionship and sexual gratification

This theme describes how internal factors such as the need for companionship and satisfaction of the sex drive interact together to make men in the current study always desire to have women by their side, even though they may have had a previous negative marriage experience. Such motivations are seen to be innate and related to personal desires and characteristics. As indicated by a respondent:

*Being a man and at my age, I cannot stay alone without a woman. Beyond the satisfaction of my sensual urges, I need a woman to provide me with domestic services; wash my clothes; cook for me to eat; keep my house tidy; etc. (Mike, 30-year-old father of five with four different women)*

As previously alluded to, this demand and the failure of certain women to meet it led some of the fathers in our study to end their relationships with their first, second, or third baby mamas and begin seeing other women.

## Discussion

This qualitative study examined the factors that drove men in Prampram, Ghana, to have children with numerous women, a phenomenon referred to as "multi-partner fertility." For the study, "serial fathering" refers to the practice of fathering children born to multiple women. Serial fathering thus occurs both inside and outside of marriage. It occurs in marriages when there is suspicion of adultery, poor spousal behavior, and/or in-law intervention, all of which are grounds for divorce in Ghana.

According to the thematic findings derived from the study, it is evident that men had children with several women due to women's negative attitudes, which prompted them to regularly change partners. Hence the study proved that men's intolerance of attitudes such as disrespect and non-performance of wifely responsibilities, suspected infidelity, maltreatment of stepchildren, and alcoholism resulted in the dissolution of their relationships with women who exhibited these attitudes. This is because the patriarchal character of Ghanaian society places males in a position of control within their marriages, hence granting them certain societal privileges that women do not have. In Ghana, marriage confers uxorem privileges to the man, such that the married woman is obligated to do domestic duties like cooking, cleaning, etc., and to sexually satisfy her husband, and the avoidance of such acts leads to intolerance on the path of most men. This adds to the study's findings, which show that men in Prampram who had children with multiple partners considered respect from their partners and the performance of traditional roles of Ghanaian women as caregivers to be critical for the sustenance of their relationship with them and continued childbearing with the woman. The absence of these, therefore, led them to move on and have children with other women. The results were in line with the study by Van Hedel et al. [35].

Some fathers, according to the study, also felt the need to move on to new relationships because they were concerned that the vices that their current spouses were involved in would be picked up by their children. To prevent that from happening, therefore, they chose to opt-out of those relationships. Also, although some fathers had children with their current spouses, they still regarded the children from their previous marriages as very important to them and would not allow them to be maltreated by their current spouses. If current spouses did not show affection and love to their stepchildren, the men did not hesitate to end such relationships and move on to others if necessary.

Women in Ghana are typically expected to be more faithful to their spouses than men. As Johnson and Young [43] argue, a woman suspected of infidelity runs the risk of losing her marriage, but the same law does not apply to men. A woman's involvement in alcoholism is likewise culturally taboo, and such women are avoided by men since they are regarded as a disgrace, and they are also scared of such behaviors negatively affecting the children they have with such women. This result was consistent with Matebese et al. [67] study. The findings further acknowledged that fathers who are close to their children are sensitive to the quality of care they receive and, as a result, will guarantee that their children receive the utmost affection and care from their caregivers. In the absence of this, such fathers may take severe actions, such as abandoning their relationship with a woman who refuses to love and care for their children since they are not the woman's biological children. This result is consistent with the findings of the studies that were conducted by [22, 53].

The study also argues that strained in-law relationships also prompt men to regularly change partners. This is because, in Ghana, marriages are between families; it is not only the man and woman who unite for this purpose [68]. Families have a crucial role in the preparation for and maintenance of marriage [69]. Hence, to prevent the dissolution of marriages, families not only approve of prospective partners but also mediate marital issues. Therefore, if in-laws disapprove of the union, it becomes difficult, if not impossible, for the couple to maintain their relationship, even if they are ready to do so. Thus, strained in-law relationships have contributed to the dissolution of marriages in Ghana and other Sub-Saharan African nations, a circumstance that pushes men to form new relationships and father children without women. This is also in line with the study from [70].

Among the Gas in Ghana, the duolocal residential system is followed, in which the man and his wife continue to live in separate family residences after marriage [7]. At her husband's request, the woman only visits the man with meals she has prepared for him, cleans the house, washes his clothes, and spends the night with him. Due to the flexibility afforded to both men and women to have several sexual partners, this living arrangement leads to a significantly greater frequency of promiscuity among the population. According to studies conducted among Ga, this residence style also contributes to the prevalence of teen pregnancy among Ga [71–73]. As a result, the people's duolocal residence pattern—a practice unique to the Gas of Ghana—as reported by this study provides a favorable environment for men to participate in multi-partner fertility and become serial fathers even when they are on the wrong side of their marital partners. The findings further state that fathers who did not share the same residence with their spouses reported that they found it easy to get into other relationships based on this residential arrangement. Hence, it was identified that this is a common practice in Prampram for couples to live apart, as is characteristic of the Ga-Adangme.

The results of the study also identified that the non-observance of marriage rites can also cause people to lose their spouses. This is because, in Ghana, marriages are not considered valid unless all marriage rights required for legal recognition have been accomplished [74]. The various ethnic groups have certain conditions that a man must meet for the union to be considered genuine. Hence, when a man does not fully fulfill the marriage ceremony, the bride's or wife's family may request custody of their daughter until the man completes the marriage payments. This is in accordance with the study conducted by [75–77]. The study further acknowledges that when women enter into consensual unions with men who have not begun or completed the customary marriage rites required to be recognized as a married couple in contemporary Ghanaian society, micro-level factors such as the need for survival, the need for a safe haven and the influence of a love charm are the driving forces. It does not, however, imply that such relationships are socially acceptable. Thus, social structural factors such as family influence prohibit such couplings, resulting in serial fathering. The findings from the study show that in Prampram, it is customarily expected of a man to first give notice (si womi) to the family of the lady they intend to marry by providing two bottles of champagne plus one hundred Ghana cedis (Gh₵ 100). This is followed by knocking (Agbo simi), which requires the man to present to the family of the woman two (2) bottles of champagne, one (1) crate of malt, and one (1) bottle of Akpeteshi plus five hundred cedis (Gh₵ 500). The man is then required to carry on with the request (sibimi) and acceptance (kplemi), for which they must provide two (2) bottles of champagne plus two hundred Ghana cedis (Gh₵ 200.00) respectively. According to the men who participated in the study in Prampram, this was difficult for them to achieve due to their low wages, which made them unwilling to marry the woman with whom they had children. This is consistent with numerous studies cited in the present study, which show that a man's financial standing determines whether he would father children with multiple women and that men with higher salaries are more likely to marry and establish happy families [47, 51, 52]. Poor men have more children [53]. They procreate with several partners to confirm their masculinity, resulting in unstable relationships.

First-birth age and other related factors have also been identified as factors that contribute to men having multiple partners. The findings from the study show that the early onset of childbearing facilitates multi-partner fertility among men in Prampram. This is because the study shows that about 10% of the fathers in this study stated that they started giving birth at an early age and at a time when they were not prepared to handle the responsibilities that came along with having children. These findings concur with the studies from [1, 30, 45, 53]. These results all support the findings of the current study, which show that the practice of serial fathering is influenced by first birth age in addition to other factors, such as low educational attainment and low income as a result of the early start of fathering. These factors all contribute to the environment in which men father children with multiple women.

Finally, the persistent need for companionship and sexual fulfillment was also identified as the reason why men have multiple partners. According to the findings of the study, although men may have had unpleasant experiences with marriage in the past, they still want women by their side for the company and the fulfillment of their sex drives. Additionally, this was a result of personal preferences. In Ghana, men usually look to women for assistance when it comes to providing domestic services, making them reliant on women for such services. As a result, when their wives are unable to provide these services, they turn to other women, whom they occasionally impregnate and end up using to father their children. According to the findings of the study, men's continual need for companionship and sexual gratification significantly influence their likelihood of having multiple children. This is in line with the findings of the study by [79–81].

### Practical implications

The study has implications for family heads in Ghana as well as for family counsellors and therapists. When providing men with counselling services on having several baby mothers, family counsellors and therapists should consider incorporating the partners of their male clients. Additionally, the counsellors should lead both spouses through pre-planned sessions for attitude improvement. Furthermore, the family counsellors and therapists should make every effort to inform men of the need to form the habit of performing obligatory marriage rites for their partners. The family heads in Ghana ought to counsel the spouses of these men to develop the practice of getting along well with their in-laws. The men's families should also counsel them to exercise more patience in their relationships, especially when young children are involved. In the long run, social services such as health education in primary and junior secondary schools increased access to family counselling services, and increased access to reproductive health services in Prampram should also be made available. Future policies and interventions aimed at reducing the prevalence of serial fathering among men in Ghana should include sexual education in peri-urban communities.

## Conclusion

This study employed qualitative approaches to examine the factors that lead men to father children with multiple women. Because of the unfavorable attitudes of women in relationships, the duolocal pattern of residence strained in-law relationships, the failure to perform marriage rites, the first-birth age, and the constant desire for companionship and sexual gratification, it was discovered that men ended up having different baby mamas. On the surface, it could seem that these men made a mainly deliberate and planned decision to practice multi-partner fertility. However, a close look at these choices in light of the outlined factors shows that certain circumstances in which the men found themselves influenced their actions. Although the men admitted that they had hoped to have long-lasting relationships with their spouses, the aforementioned issues had hindered them from doing so. This explains why they initially began having children with them. We acknowledge the role that individual attributes play in the tendency toward serial fathering and come to the conclusion that both situational and personal factors contribute to its occurrence. The limitation and avoidance of serial fathering will depend on an individual's capacity to avoid and/or control such factors. While social and cultural norms may not always push men into serial fathering, paternal multi-partner fertility or serial fathering appears to be a way out of difficult situations for them. As a result, serial fathering occurs both inside and outside of marriage. It happens in marriages when there is suspicion of adultery, poor spousal behavior, and/or in-law intervention, all of which are grounds for divorce in Ghana. It happened among the unmarried because of early childbearing, a lack of marriage rites, and a persistent demand for sexual fulfillment, which occasionally led to unintended pregnancies.

## Data Availability

The datasets generated and/or analysed during the current study are available from the author (Jemima Akweley Agyeman-jemima_ocansey@yahoo.co.uk) on reasonable request as this is part of an MPhil work.
